# Biodiesel Co-Product (BCP) Decreases Soil Nitrogen (N) Losses to Groundwater

**DOI:** 10.1007/s11270-013-1831-7

**Published:** 2014-01-05

**Authors:** M. A. Redmile-Gordon, E. Armenise, P. R. Hirsch, P. C. Brookes

**Affiliations:** 1 Rothamsted Research, Harpenden, Herts AL5 2JQ UK; 2 Sustainable Soils and Grassland Systems, Rothamsted Research, Harpenden, Herts AL5 2JQ UK

**Keywords:** Nitrate leaching amendment, Soil organic matter dynamics, Nitrification inhibitors, Straw residue incorporation, NO_3_, Biofuels

## Abstract

This study compares a traditional agricultural approach to minimise N pollution of groundwater (incorporation of crop residues) with applications of small amounts of biodiesel co-product (BCP) to arable soils. Loss of N from soil to the aqueous phase was shown to be greatly reduced in the laboratory, mainly by decreasing concentrations of dissolved nitrate-N. Increases in soil microbial biomass occurred within 4 days of BCP application—indicating rapid adaptation of the soil microbial community. Increases in biomass-N suggest that microbes were partly mechanistic in the immobilisation of N in soil. Straw, meadow-grass and BCP were subsequently incorporated into experimental soil mesocosms of depth equal to plough layer (23 cm), and placed in an exposed netted tunnel to simulate field conditions. Leachate was collected after rainfall between the autumn of 2009 and spring of 2010. Treatment with BCP resulted in less total-N transferred from soil to water over the entire period, with 32.1, 18.9, 13.2 and 4.2 mg N kg^−1^ soil leached cumulatively from the control, grass, straw and BCP treatments, respectively. More than 99 % of nitrate leaching was prevented using BCP. Accordingly, soils provided with crop residues or BCP showed statistically significant increases in soil N and C compared to the control (no incorporation). Microbial biomass, indicated by soil ATP concentration, was also highest for soils given BCP (*p* < 0.05). These results indicate that field-scale incorporation of BCP may be an effective method to reduce nitrogen loss from agricultural soils, prevent nitrate pollution of groundwater and augment the soil microbial biomass.

## Introduction

### Nitrogen management

Nitrogen (N) pollution in groundwater represents a major threat to ecology, the environment and economy (Galloway et al. 2004). In financial terms, management of ecological issues arising from aqueous reactive N pollution were estimated to cost the EU around €30 billion per annum (Sutton et al. 2011) of which the greatest contributing species is nitrate (NO_3_). Although the direct threat to human health of drinking NO_3_ contaminated water is hotly debated (van Velzen et al. 2008), regional water authorities are required to meet strict quality standards. During the industrial removal of NO_3_ from drinking water, widespread emission of N_2_O occurs (Gong et al. 2012). Besides losses through water treatment, denitrification of NO_3_ that leaches to natural systems (e.g. rivers, aquifers and subsoils) contributes extensively to indirect N_2_O emissions. Total N_2_O-N emitted to the atmosphere which is derived from NO_3_-N in solution has been estimated to account for as much as 5 % of the total N originally applied as fertiliser (Crutzen et al. 2008).

Problematically for current strategies addressing climate change, the atmospheric burden of this N_2_O can outweigh the carbon-budget benefits of displacing some dependence on fossil fuels with biofuels like biodiesel (Smith et al. 2012). Better management of N to prevent leaching of NO_3_ (and subsequent denitrification) is therefore likely to become an increasingly important goal in agricultural sustainability, with measurable impact for national sustainability. Since current management of arable and grassland soils contributes greatly to N pollution, decreasing losses from agricultural soils is also a European requirement addressed by the EU Water Framework Directive.

Leaching of N from soil in temperate arable systems is greatest in autumn/winter following harvest. It occurs as a result of concomitant factors such as low evaporation, high microbial activity, precipitation and the absence of a growing crop (Di and Cameron 2002). Towards the end of summer, microbial activity is usually limited by water availability. As rainfall increases, the still warm soil starts to moisten and microbial activity is stimulated. The subsequent mineralisation of residual organic material produces a large flush of NO_3_-N, with up to 150 kg N ha^−1^ potentially leached over the winter period (Weinert et al. 2002). Whereas direct leaching of unused fertiliser N is normally low (Macdonald et al. 1989), the large amounts of N leached from this mineralising organic material has become a characteristic trait of annual cropping systems (Sieling and Kage 2006).

### Existing Strategies to Reduce Aquatic N Loss from Soil

Existing strategies to minimise the NO_3_-N leaching problem include: legislative restrictions on the timing and quantity of fertiliser application; reducing the period that a soil remains fallow; the use of cover crops and incorporation of residues. Cover-crops or ‘catch-crops’ typically prevent less than 50 % of leaching (Justes et al. 1999; Sieling and Kage 2010). These require ploughing back into the soil in spring, whereupon 20 to 55 % of the immobilised N is made available to the succeeding crop (Malpassi et al. 2000). However, asynchrony of the management system can result in lower yields due to prolonged N immobilisation, and soils are frequently too wet for the cover crop to be re-incorporated at the correct time (Richards et al. 1996; Vyn et al. 1999; Griffin et al. 2000). Although cover crops which are killed by autumn frosts are not subject to these limitations, this strategy is less effective in preventing N leaching (Weinert et al. 2002). As an alternative to spring incorporation of green cover crops, herbicides can be applied, but this is costly and frequently ineffective (Fisk et al. 2001).

A single incorporation of agricultural residues to soil after harvest can also immobilise some NO_3_-N, but this strategy is generally less effective than using cover crops (Justes et al. 1999) and also results in unpredictable duration of immobilisation (Thomsen and Christensen 1998). Use of cover crops and/or incorporation of arable residues to soil can also increase the risks of both introduction and seasonal carry-over of phytopathogens (Kumar and Goh 2000). Incorporation of exogenous materials to soil avoids the problems associated with pathogen persistence. Vinten et al. (1998) found that incorporation of waste paper showed good potential to immobilise soil-N, but repeated annual applications increased the concentrations of arsenic and nickel in the soil, and were thus unsuitable for agriculture. Rahn et al. (2009) showed that cardboard waste (combined with sugar-beet residues) could be a more favourable option, preventing about 40 % of N leaching compared to controls. Alternative approaches, such as the use of nitrification inhibitors can be effective in lowering nitrate leaching and N_2_O emissions (Menendez et al. 2012), but these are expensive and do nothing to immobilise the NO_3_-N pool already present in the soil at the time of application.

### An Alternative Approach

Biodiesel is an alternative fuel derived from the transesterification of animal or vegetable oils. The sustained rise in biodiesel production worldwide since around 2005 has created a surplus of glycerol, which forms the bulk of the waste product associated with biodiesel production (Manosak et al. 2011). In Europe alone, a surplus of more than 6 × 10^5^ metric tonnes of liquid waste was produced in 2007 (André et al. 2010). Research activities attempting to identify potential uses for the glycerol fraction of this waste are abundant and diverse, ranging from direct use in polymer compositions (Rosa et al. 2010) or animal feeds (Lage et al. 2010) to a substrate for microbial production of hydrogen by *Enterobacter* sp. (Nakashimada et al. 2009), 1,3-propanediol by *Clostridium* sp. (Papanikolaou et al. 2008) and Omega-3 fatty acids by *Schizochytrium* sp. (Ethier et al. 2011). Many of these existing uses require a high purity of glycerol (>97 %) whereas the co-product from biodiesel production (BCP) usually consists of around 60 % glycerol (Zhou et al. 2008), being a mixture of methanol, water, potassium and/or sodium salts, soaps, residual biodiesel, fatty acids and traces of unreacted mono-, di- and triglycerides (Thompson and He 2006; Kongjao et al. 2010). Purification of BCP to extract glycerol of sufficient purity is often prohibitively expensive (Zhou and Boocock 2006), whereas the initial step of recovering the excess methanol by distillation is usually economically favourable, with this methanol often being re-used to make more biodiesel (Raghareutai et al. 2010).

We hypothesised that BCP could be applied to the soil to cause increased immobilisation of NO_3_-N by the soil microbial biomass. If more effective than traditional approaches, the proposed management could provide multiple beneficial impacts for the environment and agriculture, and therefore the efficiency of biodiesel production. The effects of BCP incorporation on N leaching and total microbial biomass were therefore compared with those of milled grass and cereal straw incorporation.

## Materials and Methods

### Overview

Application of de-methylated (otherwise unrefined) BCP to soil was hypothesised (1) to increase immobilisation of NO_3_-N by the soil microbial biomass, and furthermore (2) that the effect would be greater and more rapid than traditional approaches using plant-residue incorporation. This was investigated in a series of three experiments. Experiment 1 was a preliminary study to determine if incorporation of BCP affected extractable NO_3_-N and total microbial biomass, and to establish an approximate response to application rate. Experiment 2 traced the NO_3_-N, NH_4_-N, organic-N and microbial biomass-N dynamics over time following application of BCP. Experiment 3 compared N leaching between BCP and crop residues in an arable soil over a winter period typical of Northern Europe. This was conducted in ‘semi-natural’ conditions, i.e. mesocosm-lysimeters in the open air environment.

### Soil Sampling and Preparation

Three soils were sampled from three long-term experiments at Rothamsted Research, Hertfordshire, UK (50°50′ N, 0°25′ W). The soils’ main characteristics are reported in Table 1. All soils were Chromic Luvisols.

**Table 1:** Soil properties

Soil no.	Name	Experiment no.	Organic C (g kg^−1^)	Total N (g kg^−1^)	C/N ratio	pH	Extractable inorganic N (mg kg^−1^ soil)
1	Hoosfield	1	9.2	0.98	9.39	5.00	56.4
2	Highfield	2	19.9	1.92	10.36	6.76	110.6
3	Long Hoos	3	13.66	1.30	10.51	7.18	19.8

Soil 1 was obtained from the long-term Hoosfield experiment which received a single dressing of chalk (150–250 t ha^-1^) in the nineteenth century. Since then it has not received any other amendment, including chemical or organic fertiliser. Hoosfield is a flinty, silty clay loam, classified as Batcombe Series (Avery 1980) and was sampled in February, 2008. Soil 2 was a fine silty loam over clayey drift, taken from the cereal rotation of the Highfield Ley-Arable Experiment (Johnston et al. 2009), again classified as Batcombe Series (Avery 1980) and sampled in June, 2009. Soil 3 was obtained from the ‘Long Hoos’ site, which has been under long-term arable rotations since the 1950s or earlier. The soil is a flinty clay loam over clay with sand inclusion (Batcombe-Carstens series; Avery 1980) sampled in November, 2009. All soils were collected using a 2.5-cm auger to a depth of 0–23 cm, in a ‘W’ pattern across the sites. The bulked cores were stored overnight at 10 °C, and then sieved moist <2 mm. The soils were subsequently adjusted to 40 % of the water holding capacity (WHC) and soils 1 and 2 were stored at 25 °C in the dark for 7 days to encourage mineralisation of organic N to NO_3_-N before treatments were applied. Soil 3 was processed at 10 °C with no pre-incubation period.

### Production of Biodiesel Co-Product

Waste vegetable cooking oil was obtained from a number of local restaurants and bulked before use. The BCP was produced from a small-scale (100 L) batch reactor. Biodiesel and BCP were produced by mixing CH_3_OH (methanol) and KOH (potassium hydroxide) to give 5 and 0.15 M L^−1^ oil, respectively. The methanol and potassium hydroxide were mixed before introducing into the preheated oil (60 °C) as a steady stream for 5 min. This was continuously circulated using a centrifugal pump set to 60 L min^−1^ for 1 h. In addition to the 0.15 M KOH L^−1^ required as a catalyst, a further 0.04 M L^−1^ was added to neutralise free fatty acids in the waste oil (determined by titration of waste oil against a standard of aqueous KOH (0.018 M) together in propan-2-ol with a phenolphthalein indicator). After pumping, the mixture was allowed to settle for 24 h before separating the fuel phase (biodiesel) from the co-product (BCP). The BCP was purged of excess methanol by heating at 90 °C for 2 h. The pH of the BCP was measured after dissolving 20 % in aqueous solution (*w*/*v*).

Excess methanol was removed from the BCP at atmospheric pressure by heating 1 L of BCP in a 5-L capacity beaker on a magnetic stirring hot plate at 90°C. There was no significant volume change after 2 h of heating. Upon cooling the resulting mixture became a highly viscous black liquid (ca 20 % loss *v*/*v*). This BCP was dissolved in water to make a 20 % solution (*w*/*v*) for application to soil.

### Experimental Design

#### Experiment 1:

Three replicates of moist soil containing 100 g dry weight soil 1 were mixed with BCP at rates of 150, 500 and 1500 μg BCP-C g^−1^ soil (Table 2) and incubated in plastic bags in the dark at 25 °C for 1 week. At the end of the incubation, soils were analysed for NO_3_-N content and microbial biomass N (Section 2.6).

**Table 2:** Treatments

Experiment	Soil	Substrate types	Substrate concentration (μg C g^−1^ soil)	Co-application of NH_4_NO_3_ (μg N g^−1^ soil)	Temp. (°C)
Experiment 1	Soil 1	BCP	0, 150, 500, 1500	0	25
Experiment 2	Soil 2	BCP	0, 1500	0, 50	25
Experiment 3	Soil 3	Milled meadow grass, milled wheat straw, BCP	0, 1500	0	Variable (ref Fig. 1)

#### Experiment 2:

The results from experiment 1 formed the basis of subsequent application rates. Three replicates of BCP were added to soil 2 at 1500 μg C g^−1^ soil, and incubated as above for 25 days (controls received only water). Ammonium nitrate (NH_4_NO_3_) was co-applied with BCP (and not) at a rate of 50 μg N g^−1^ soil; controls received none. Subsamples of 50 g were taken from each mesocosm and divided into two: fumigated and non-fumigated, firstly at 4 h, then at 4, 11 and 25 days after substrate addition to assess K_2_SO_4_ extractable NO_3_-N, NH_4_-N, organic-N and total microbial biomass N.

#### Experiment 3:

**Fig. 1:**
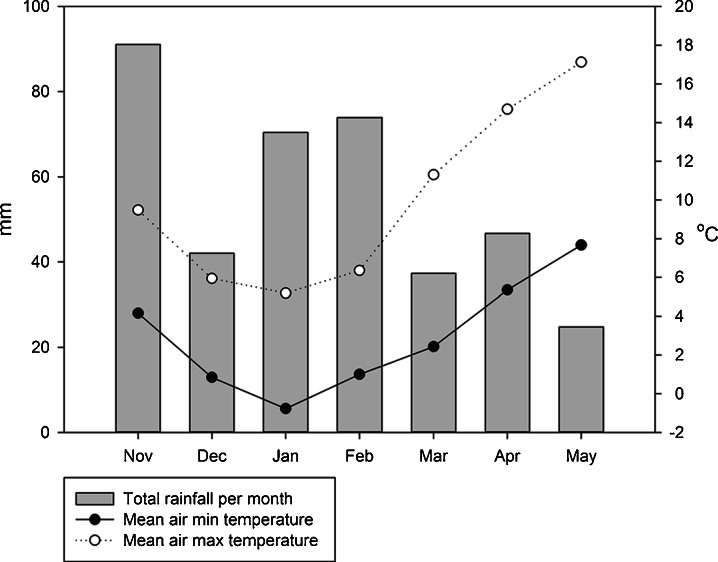
Meteorological data from Autumn 2009 to Spring 2010 (local to experiment 3)

Four treatments were compared using soil 3. Treatments included three substrate additions, and one control. Treatments were: (1) control, (2) milled meadow grass (from the Park Grass Continuous Hay Experiment, containing 47.22 % C and 1.92 % N; (3) milled wheat straw: 46.36 % C, 0.559 % N and (4) BCP: 61.04 % C, 0.041 % N, pH 9.76. Substrates were mixed with the soils at a rate of 1.5 g substrate-C kg^−1^ soil (dry weight). The soils were then packed into tubular mesocosms as described in Section 2.5, and were thereby subjected to the prevalent weather conditions in the southeast of England from November 2009 to May 2010 (Fig. 1). Across this period, leaching losses were collected immediately after each rainfall event and the total leachate volume was recorded. Any germinating weeds were removed and discarded as soon as they appeared. The total drainage water from each leaching event was filtered through a Whatman no. 42 filter paper, and a 40-mL representative sample stored at −20 °C prior to analysis of total dissolved organic carbon (DOC), inorganic N (NO_3_-N and NH_4_-N), and total N. After 162 days, the mesocosms were destructively sampled for measurements of soil microbial biomass C, ATP and N (Section 2.6).

### Lysimeter Design (experiment 3)

Open-topped mesocosm–lysimeters were constructed using 30 cm lengths of uPVC pipe, 11 cm in diameter. Each tube was fixed to a high-density polyethylene funnel filled with acid-washed gravel to support the soil and enable drainage. A nylon mesh (1 mm) was placed at the soil/gravel interface. Each mesocosm-lysimeter was packed with 2 kg of fresh soil (40 % WHC) inclusive of treatment to a bulk density of 1.1 kg L^−1^. Fine quartz sand was used to fill the void between the four mesocosm tubes within each box to buffer against fluctuations in temperature. Each mesocosm was positioned above a 250-mL dark glass jar, into which the leachate was collected. Each block of four mesocosms was then placed in an open mesh cage exposed to natural fluctuations in rainfall and temperature (Fig. 1).

### Soil Chemical and Microbiological Analyses

Soil total C and N concentrations were determined on finely ground soil samples by dry combustion using a LECO CNS-2000 auto-analyser. Soil pH was measured at a 1:2.5 soil/water ratio with a glass electrode. Inorganic N (NO_3_-N and NH_4_-N) of soil extracts and leachate was quantified by automated photometric procedures based on cadmium reduction using a Skalar SANPLUS Continuous Flow Analyser. Total N in water samples and extracts was measured by manual persulfate oxidation of inorganic and organic-N to NO_3_-N (Cabrera and Beare 1993) and subsequently reanalysed with the continuous flow analyser as above. Total dissolved organic C was measured using a high-temperature Fermanox TOC analyser.

Soil microbial biomass C was determined by fumigation–extraction using moist soil containing 50 g oven dry weight equivalent (Vance et al. 1987; Wu et al. 1990). From the same extracts, soil microbial biomass N (BN) was estimated from the difference between extractable N (EN) from the fumigated and unfumigated soils (EN_diff_) corrected according to Sparling et al. (1996): BN = 2.22 EN_diff_. Organic N was calculated by subtracting inorganic N from the total. Soil microbial biomass ATP was measured according to the method of Jenkinson and Oades (1979) as modified by Redmile-Gordon et al. (2011).

At the end of the lysimeter study (experiment 3) N mineralisation (determined as ‘potentially mineralisable N’, PMN) was investigated by aerobic incubation (Stanford and Smith 1972). Fresh, moist soil (50 g oven dry weight equivalent) was mixed with 50 g of acid-washed quartz sand, which had previously been heated at 500 °C for 6 h. The mixture was then placed in glass leaching tubes fitted with sintered glass frits to retain soil (total capacity approximately 100 ml). These sub-samples were incubated at 25 °C for 6 weeks. After 0, 2, 4 and 6 weeks, each glass lysimeter was flushed with 50 ml of 0.01 M CaCl_2_. To maintain mineralisation, 25 mL of N-free nutrient solution was added after each flush to replace nutrients removed from the soil during leaching (Carter and Gregorich 2008). The NO_3_-N and NH_4_-N concentrations were then determined as described previously.

### Statistical Analyses

One-way analysis of variance was conducted to determine substrate type effect on soil chemical and microbiological parameters (experiment 3). The means were compared using Fisher’s least significant difference (LSD) test. All statistical analyses were performed using R (Team 2011).

## Results

### Experiment 1: Investigating the Potential of BCP for NO_3_-N Immobilisation

**Fig. 2:**
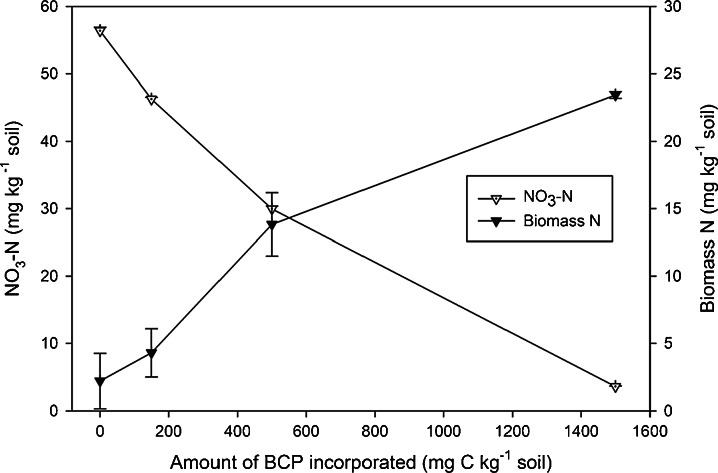
K_2_SO_4_ extractable NO_3_-N, and biomass-N, 7 days after BCP application (experiment 1)

Seven days after incubation, extractable NO_3_-N in the control soil was 56.4 ± 0.5 mg N kg^−1^ soil. This was decreased by increasing the amount of BCP incorporated (Fig. 2). A mean decrease of 10.1 mg N was achieved with the 150 mg BCP-C treatment, 26.4 mg N with 500 mg BCP and 52.8 mg N with 1500 mg BCP-C. These decreases in NO_3_-N were coupled with increased BN, which accounted for approximately half the N immobilised (Fig. 2). Ammonium N concentrations (not shown) were low and unaffected, ranging from about 2.2 to 4.2 mg NH_4_-N kg^−1^ soil. The highest application rate (1500 mg BCP-C kg^−1^ soil) caused the greatest apparent immobilisation, and was applied in both subsequent experiments.

### Experiment 2: NO_3_-N, NH_4_-N, Organic-N and Microbial-N Dynamics

**Fig. 3:**
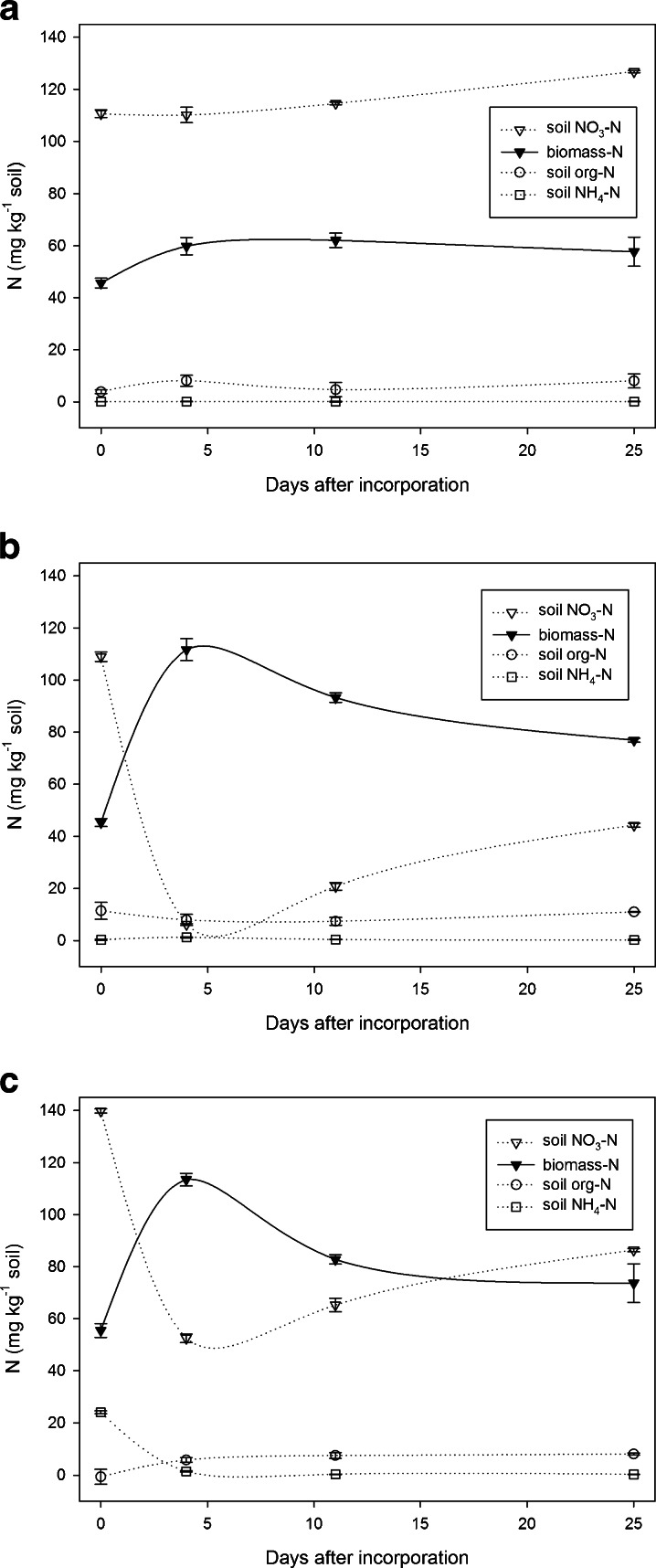
Changes in soil-N and biomass-N at 25 °C shown for **a** control soil; **b** soil + BCP; **c** soil + BCP + 50 mg NH_4_NO_3_-N (experiment 2)

Four hours after application of BCP treatments to soil 2, NO_3_-N concentrations were 110.6 ± 1.5, 109.0 ± 1.8 and 139.8 ± 0.8 mg N kg^−1^, for control soil (Fig. 3a), soil given BCP (Fig. 3b) and BCP plus N (Fig. 3c), respectively. Corresponding microbial biomass N concentrations were 45.7 ± 1.9, 45.4 ± 1.5 and 55.5 ± 2.7 mg N kg^−1^ soil, respectively. Organic N and NH_4_-N concentrations remained low and stable in all treatments, at all times, except for the first measurement of soil given BCP co-applied with N fertiliser (50 mg NH_4_NO_3_-N): at 4 h, 29.2 mg more NO_3_-N and 23.9 mg more NH_4_-N were measured compared to the control (53.1 mg more inorganic-N kg^−1^ soil).

After 4 days, soil given BCP had a markedly decreased extractable NO_3_-N content of 6.2 ± 0.3 mg N kg^−1^, whilst biomass N had increased to 111.7 ± 4.2 mg N kg^−1^ (Fig. 3b). In soils given BCP and N (Fig. 3c), extractable NO_3_-N was 52.5 ± 1.7 mg kg^−1^ soil, whilst biomass N was 113.5 ± 2.4 mg N kg^−1^ soil (similar to soil given BCP without N). The BCP treatment, in both cases, was sufficient to lower soil NO_3_-N by approximately 100 mg NO_3_-N kg^−1^ soil (a decrease of 66.7 mg NO_3_-N g^−1^ BCP-C).

From 4 days onward, N in the control soil was mineralised at a slow and steady rate (0.8 mg N kg^−1^ soil day^−1^; Fig. 3a). Biomass N was always lowest in the control. After this time the biomass N concentration in the ‘BCP’ and ‘BCP plus N’ treatments began to decline at mean rates of 1.7 and 1.9 mg N kg^−1^ soil day^−1^, respectively. This was mirrored by a change in the N mineralisation rates, which became positive in both BCP treatments: increasing to 1.8 and 1.6 mg N kg^−1^ soil day^−1^, respectively (almost double the rate of the control). The NO_3_-N and biomass N pools thus showed closely coupled inverse dynamics (see Fig. 3b and/or Fig. 3c).

### Experiment 3: Efficacy of Immobilisation in Exposed Conditions

#### Cumulative Inorganic N in Leachate

**Fig. 4:**
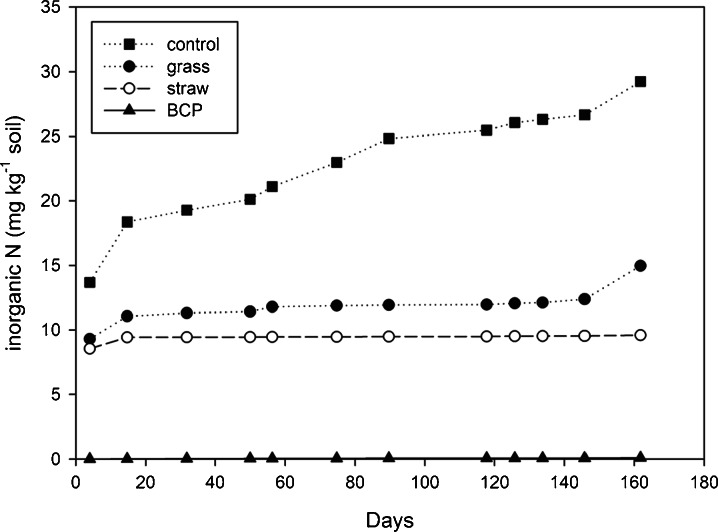
Cumulative inorganic N leached (experiment 3)

In conditions of fluctuating temperature and stochastic rainfall, total inorganic N loss to water from the control soils (soil 3) was 29.3 mg N kg^−1^ soil. In contrast, soils treated with BCP lost only 0.1 mg N kg^−1^ soil as inorganic N over the same period (Fig. 4; November 2009 to May 2011). BCP thus prevented more than 99 % of inorganic N from leaching. Incorporation of finely milled wheat straw and meadow grass decreased the total inorganic N leached by 19.7 and 14.3 mg N kg^−1^ soil (67 and 49 %, respectively).

#### Cumulative Total N in Leachate

**Fig. 5:**
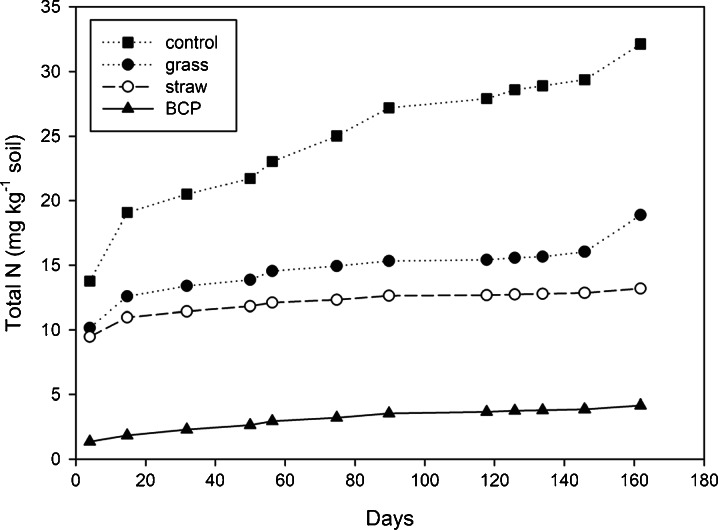
Cumulative total N leached (experiment 3)

Biodiesel co-product also significantly reduced total N leaching (sum of organic- and inorganic-N). Total N loss to water from the control soils was 32.1 mg N kg^−1^. Only 4.2 mg N kg^−1^ soil was leached over the entire period from soils treated with BCP (Fig. 5). Total leaching of N from grass and straw treatments was 18.9 and 13.2 mg N kg^−1^ soil, respectively.

#### Cumulative Organic C in Leachate

**Fig. 6:**
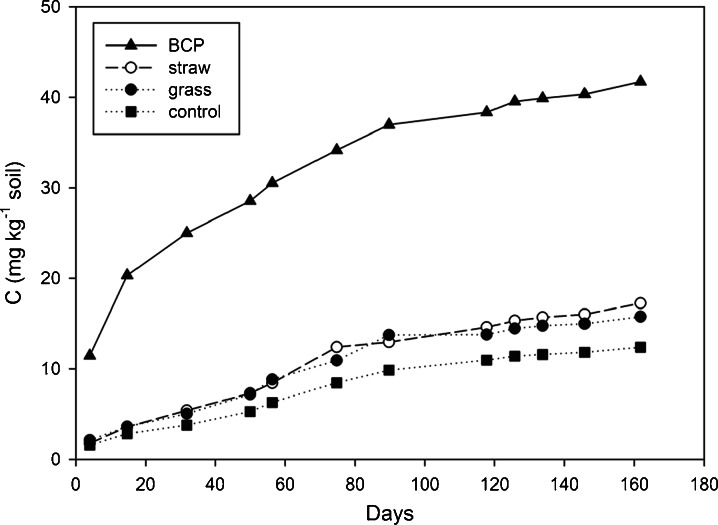
Cumulative DOC leached (experiment 3)

Mesocosms treated with 1500 g BCP-C kg^−1^ soil, leached only 30 mg more organic C, over the entire period of measurement compared to controls (Fig. 6). Incorporation of wheat straw and grass at the same rate increased C leaching by approximately 5 mg organic C kg^−1^ soil. Leaching of organic C was greatest during the first month after BCP application. Then DOC leaching rates became similar for all soils.

#### Soil Microbiological Characteristics

**Fig. 7:**
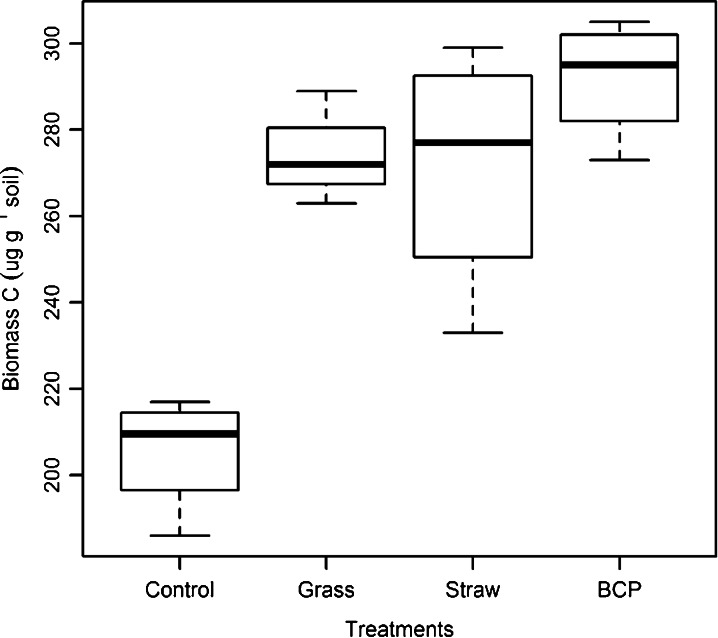
Microbial biomass C, 6 months after substrate incorporation (experiment 3)

**Fig. 8:**
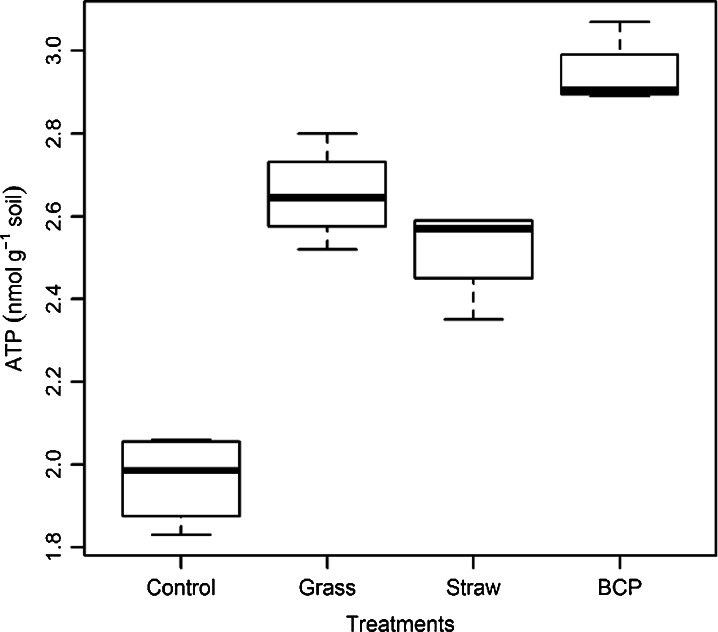
Microbial ATP, 6 months after substrate incorporation (experiment 3)

At the end of the leaching period, 162 days after substrate addition, biomass C was lowest in the control soil, and highest in the BCP treatment (Fig. 7). Soil ATP concentrations indicated a markedly larger microbial biomass in soil treated with BCP (Fig. 8). The difference in microbial biomass was greater when using ATP as an indicator (and statistically significant over all other treatments; *p* < 0.05). When microbial biomass was quantified by biomass C, the measured increase from BCP was not statistically distinct from either grass or straw treatments (LSD 27.94 mg biomass C kg^−1^ soil; Table 3). Biomass N was lowest in the control soil, but highest for the grass treatment, with no statistically significant differences between soils that had received substrate (LSD 7.53 mg biomass N kg^−1^ soil; Table 3).

**Table 3:** Properties of the microbial biomass

Treatment	Biomass C concentration (mg kg^−1^)	Biomass N concentration (mg kg^−1^)	Biomass C/N ratio	Biomass ATP (nmol g^−1^ soil)	Biomass ATP concentration (μmol ATP g^−1^ biomass C)
Control	205.5 b	25.62 b	8.0	1.97 c	9.56
Grass	274.0 a	38.14 a	7.2	2.65 b	9.68
Straw	271.5 a	36.96 a	7.4	2.52 b	9.28
BCP	291.9 a	35.75 a	8.2	2.94 a	10.08
ANOVA F pr.	<0.001	0.013		<0.001	
SEM^a^	9.07	2.45		0.0536	
LSD^b^	27.94	7.53		0.165	

Means with the same letters are not statistically different at the 5 % confidence level

^a^Standard error of the means

^b^Least significant difference calculated at the 5 % confidence level

#### Soil Organic Matter

All treatments significantly increased soil organic C relative to control soils (*p* < 0.001; Table 4). Total N in the control soil, 162 days after incorporation, was 1,284 ± 6 mg N kg^−1^ soil (*n* = 4). All incorporations also significantly increased total N in the soil at this time (LSD 16.51 mg kg^−1^ soil; *p* < 0.05). The grass treatment resulted in the highest total soil N: 1.34 ± 0.007 g N kg^−1^, representing an increase of about 60 mg N kg^−1^ soil. However, since 61 mg N kg^−1^ soil was incorporated from the grass itself, overall there was a net loss of N. In contrast, the BCP treatment caused an increase in soil N of 37 mg N kg^−1^ soil, with less than 1 mg of N introduced in the BCP itself. Soil total N was thus increased through application of BCP, exclusive of N contained in the treatment, with a statistically significant increase relative to untreated controls (Table 4). The increases in soil N were coupled with increases in soil C, suggesting that much of the immobilised N was retained in the soil organic matter.

**Table 4:** N budget, including C/N ratio of substrate at 0 days, and soil 162 days after substrate incorporation (inc.)

Treatment	Substrate C incorporated (g kg^−1^ soil)	Substrate N incorporated (mg kg^−1^ soil)	Substrate C/N (labile fraction)	Soil total C (g kg^−1^)	Soil total N (mg kg^−1^ soil)	Soil total N difference to control mean (mg kg^−1^ soil)	‘N efficiency’ (soil total N difference minus substrate N incorporated) (mg kg^−1^ soil)	N efficiency accounted for by leaching prevention (mg kg^−1^ soil)	‘N balance’ unaccounted for (mg kg^−1^ soil)
Control	0	0.00	–	13.76 b	1,284 c	0 c	0	0	0 ab
Grass	1.50	61.00	25	14.55 a	1,344 a	60 a	−1	13.2	−14 b
Straw	1.50	18.09	83	14.43 a	1,325 b	41 b	23	18.9	+4 a
BCP	1.50	0.98	1,526	14.33 a	1,321 b	37 b	36	28.0	+8 a
ANOVA F pr.				<0.001	<0.001	<0.001			<0.042
SEM^a^				0.11	5.36	5.36			5.52
LSD^b^				0.34	16.51	16.51			17.67

Means with the same letters are not significantly different at the 5 % confidence level

^a^Standard error of the means

^b^Least significant difference calculated at the 5 % confidence level

#### Soil Potentially Mineralisable N

**Fig. 9:**
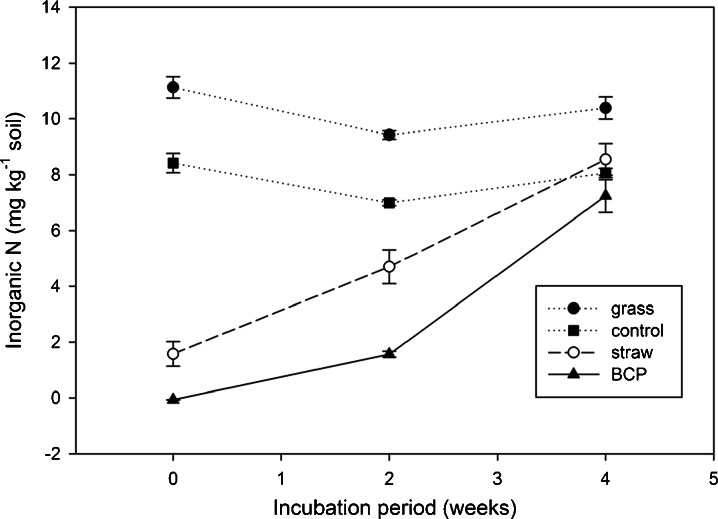
Soil re-mineralising N (soil returned to laboratory to assess PMN after external incubation; experiment 3)

The mineralisation of soil N in the different treatments from 162 days onwards, can roughly be divided into two groups (Fig. 9), viz. group 1: control and grass, and group 2: BCP and straw. In group 1 there was considerable inorganic N already present at the start of the laboratory incubation (leached at ‘incubation time 0’), ranging from 8 to about 12 mg N kg^−1^ soil. In group 2, the straw-amended soil yielded much less inorganic N than was initially present in the soils of group 1 (only about 2 mg N kg^−1^ soil), the BCP-treated soils yielded no measurable inorganic N at the start of the 25 °C laboratory incubation. By week 2, mineralisation of N had also begun in soils treated with the BCP, and between weeks 2 and 4 the mineralisation rate increased rapidly, showing the highest rate increase of all treatments.

## Discussion

In experiment 1, biodiesel co-product decreased extractable inorganic N by >50 mg NO_3_-N kg^−1^ soil (Fig. 2). There was a corresponding increase in the microbial biomass, but only half of the NO_3_-N decrease was accounted for by the increase in living biomass N. This demonstrates that the microbial biomass cannot entirely account for all of the immobilised NO_3_-N. Though it should be noted that there may be some small underestimation of both organic and biomass N in the more concentrated extracts (soils given 1500 μg BCP g^−1^ soil), this small negative bias was unlikely to account for such a large difference (Cabrera and Beare 1993), and was therefore suggestive of other pools not quantified (e.g. NH_4_-N, microbial metabolites, or non-extracted soil organic matter).

In experiment 2, extractable NH_4_-N and organic-N pools were also measured over time. Here, the BCP treatment appeared to prevent the loss of up to 100 mg NO_3_-N kg^−1^ soil upon extraction (a decrease of 66.7 mg NO_3_-N g^−1^ BCP-C). Both experiments suggested that NO_3_ losses with BCP were much smaller than typically observed using agricultural residues (Ocio et al. 1991). No toxic effect of BCP was seen 4 h after incorporation—as the biomass N and C in soils, with or without BCP, were equal (Fig. 3a and b). An eco-toxicological study by Lapinskiene et al. (2006) showed that biodiesel itself (contained in small and variable amounts in BCP) also causes no signs of toxicity when added to soil, even when incorporated at concentrations as high as 12 % *w*/*w*. In the present study, the microbial biomass increased markedly sometime between 4 h and 4 days of BCP addition. Other studies using BCP as a biological feedstock have also revealed improved growth rates, e.g. with swine given dietary supplements of up to 8 % BCP (Schieck et al. 2010). Furthermore, in experiment 2, a similar decrease in soil NO_3_-N was observed at 4 days in soils amended with BCP, with or without the simultaneous addition of NH_4_NO_3_ (Fig. 3c). This suggests that N limitation of microbial biomass did not occur.

The relationship apparent between biomass-N and NO_3_-N (Fig. 3b and c) strongly suggests that the microbial biomass is at least partly responsible for the decreased soil NO_3_-N. The greater re-mineralisation rates subsequently observed in soils given BCP suggest that the previously immobilised N was ‘naturally’ re-mineralised without the need for stimulating a positive priming effect through C addition, which was previously thought to be required (Chaves et al. 2007). This turnover of N is most likely due to dwindling reserves of available C in the soil, resulting in cell death. The stable organic and NH_4_-N pools indicate that any losses from the biomass N pool due to cell mortality are either rapidly oxidised to NO_3_-N, or quickly re-assimilated during microbial turnover. The microbial biomass in control soils was unstable. At 4 days, biomass N in the control soil increased to about 60 mg N kg^−1^. Joergensen and Raubuch (2003) considered that the microbial biomass becomes quickly and temporarily depleted of intracellular reserves shortly after soil disturbance. After sieving etc., contact between microbes and newly exposed substrate ‘hotspots’ would then stimulate microbial growth (Joergensen and Potthoff 2005). This appears to have become detectable in the control sometime between the 4 h and 4 day measurements.

In experiment 3, after exposing the mesocosm lysimeters to the weather, it rained heavily on the second and third days (17 and 16 mm, respectively). However, in the mesocosms receiving BCP, both organic and inorganic N (collected immediately after) remained negligible. In contrast, 10–15 mg N kg^−1^ soil was leached from soils with all the other substrate amendments. Such rapid and effective immobilisation of N through the application of BCP is exceptional because the mean soil temperature (7 °C at 0–10 cm) is not considered to be conducive to rapid N turnover (Stark and Firestone 1996; Xu et al. 2010). Although agricultural amendments, i.e. straw and grass, decreased some N leaching at this time (initially less than 50 % of leaching compared to controls), they were slower to reach optimum immobilisation (Fig. 4).

The delay in N immobilisation using crop residues is to be expected because these relatively complex and recalcitrant substrates take longer to metabolise (Meli et al. 2003) and thus encourage slow-growing microbes (Blagodatskaya et al. 2009). The mechanisms for the early immobilisation of N seen here for straw and grass (first sampling point) are also likely to be partly physical rather than microbiological, as some of the NO_3_ contained in soil water at the time of incorporation would have been absorbed by the dry, milled plant material. The material was added dry to ensure even mixing, and while this is normal practice in laboratory trials, the increased surface area of substrate accelerates microbial activity (Shelp et al. 2000; Magid et al. 2006). This means that N immobilisation is likely to occur more slowly when using intact residues in the field, e.g. Shen et al. (1997). In contrast, BCP was applied at the same rate and dilution as might be practical in agriculture (1500 mg BCP-C kg^−1^ soil; as a 20 % solution in water), and since it was in solution the substrate very rapidly dispersed. The possibility of direct application of BCP to the soil surface without the need for incorporation remains an important consideration, because traditional residue incorporation practices require tillage, which causes increased greenhouse gas emissions (Abdalla et al. 2013). From laboratory observations (not shown), it appears that multivalent cations in the soil exchange for monovalent cations associated with fatty acids in the BCP, causing precipitation in the upper layers of soil. The more soluble glycerol component would penetrate deeper into the soil profile. Glycerol therefore probably accounts for the small losses of DOC at the beginning of the trial. All treatments caused a small increase in leachate DOC compared to the control. The biodiesel co-product resulted in the greatest increase, with a net total of 30 mg C kg^−1^ soil being leached. When compared to the total amount of carbon added (1500 mg C kg^−1^ soil) this corresponds to a maximum loss of only 2.2 % of the initial C application (Fig. 6). Thus biodiesel co-product can be said to have a high affinity for the soil.

Extractable NO_3_-N in soil 3 (experiment 3) was initially only 20 mg kg^−1^ (Table 1). The application rate of BCP-C (1500 mg kg^−1^ soil) had the capacity to immobilise a far greater quantity of NO_3_-N than was initially available in the soil (as shown in experiments 1 and 2). Re-mineralisation only began in May after artificially raising the temperature to 25 °C as part of the PMN procedure (Section 2.6). This suggests the quantity of BCP added was too great for early mineralisation to occur. Aerobic incubation is generally considered to be the standard method to assess potential soil N supply (Zhao et al. 2009). Unfortunately, repeated vacuuming of the columns during the PMN procedure caused compaction of the soils after 4 weeks of incubation. By week 6 it was clear (from odour) that they had become anaerobic. No further meaningful data could thus be obtained from the PMN study. It is therefore recommended that future aerobic mineralisation studies with clay soils use a higher sand/soil ratio and/or avoid the vacuum method for acquisition of water samples. Re-mineralisation dynamics remain one of the great challenges facing predictive soil testing (Ros et al. 2011) and N pollution control strategies (Chaves et al. 2007).

The duration of N immobilisation in experiment 3 appeared to be much greater when the soil was completely depleted of N compared to when N was not limiting (experiment 2). The duration of immobilisation has important implications, depending on the primary soil management objective, being either (1) inorganic N release to crop in the following year, e.g. Chaves et al. (2008) or (2) longer-term soil organic matter accumulation, which is perhaps more relevant to slow climate change, e.g. Bolan et al. (2012). The duration of immobilisation was greater using BCP than milled crop residues. This is most probably because very little N was contained in the BCP treatment itself, and suggests much less BCP is required to immobilise the same quantity of N compared to straw. Soil water content fluctuated greatly during experiment 3, with prolonged dry periods in the spring. N mineralisation is highly dependent upon temperature and water content, but more work is required to enable prediction of N release dynamics in the field given contrasting amendments (Guntinas et al. 2012). Since total N leaching throughout was only marginally greater than that of inorganic N for all treatments, this data supports previous work suggesting that soluble organic N is a relatively insignificant pool of N loss in arable systems, e.g. Murphy et al. (1999).

Soil biomass C measured at the end of experiment 3 had increased in response to addition of all amendments. However, biomass C in soils recently given organic amendments should be considered with some caution, as small artefacts are suspected to occur through increased solubility of non-living organic residues during chloroform extraction (Kuzyakov et al. 2009). In such cases, a complementary and independent measure of the microbial biomass may be appropriate (Dyckmans et al. 2003; Redmile-Gordon et al. 2011). Using ATP as an independent measure of the microbial biomass, the increases due to BCP were found to be highest of all the amendments by a statistically significant margin (*p* < 0.05). Although biomass C in soils given BCP, straw and grass had increased relative to the control, differences between the amendments were not statistically significant (Table 3). This is possibly, in some small part, due to grass and straw contributing artefacts as described by Kuzyakov et al. (2009), but in the present study, this is more likely due to the greater standard error of the means associated with biomass C measurements, compared to ATP (Table 3).

If hydrophobic BCP residues had remained in the soil at the time of measurement (e.g. chloroform soluble lipids or biodiesel) then an artefactually increased measure of biomass C would result (giving an unexpectedly low biomass ATP concentration for this treatment). This did not occur, and the biomass ATP concentration was actually highest of all in soils given BCP (10.08 μM ATP g^−1^ biomass C). This sits comfortably within the range typically found in agricultural soils worldwide (Contin et al. 2001) and gives confidence that the increased biomass C, caused by BCP addition, was not an artefact of any residual organic fraction. This is also in accordance with Lapinskiene et al. (2006) who found no extractable biodiesel in soil just 2 weeks after a spillage event.

Approximately half of the N that was prevented from leaching by the addition of BCP was accounted for as microbial biomass N. The low microbial biomass C/N ratio measured after incorporation of grassland residues *may* be due, in part, to chloroform releasing some N from lipid-occluded alkaloids produced by plants or endophytic microorganisms (Zhang et al. 2012; Cripps and Edwards 2013). Other studies point towards C/N ratio of the microbial biomass as being indicative of community structure differences, such as a fungal dominated community at high C/N ratios, although there is some controversy surrounding interpretation of these studies (Strickland and Rousk 2010). It was also suggested that a higher microbial biomass C/N ratio may be explained by low microbial activity (Maithani et al. 1996) but our results suggest the opposite, with the highest microbial biomass C/N ratio coupled with the highest soil ATP content (in soil given BCP; Table 3).

Nevertheless, since overall N efficiency and N balance (Table 4) cannot be accounted for by N in the microbial biomass alone (Table 3), it appears that other pools of soil organic matter were augmented, such as ‘non-extractable residues’ (Nowak et al. 2013) or ‘necromass’ (Miltner et al. 2012; Schurig et al. 2013). In future studies the use of stable isotopes at field and catchment scales is recommended to more completely elucidate the pools and factors affecting transformations of reactive N.
